# Melanotic Neuroectodermal Tumor of Infancy: A Clinicopathological and *BRAF* V600E Mutation Study of 11 Cases

**DOI:** 10.3389/fonc.2021.668505

**Published:** 2021-05-20

**Authors:** Rong-Hui Xia, Chun-Ye Zhang, Li-Zhen Wang, Yu-Hua Hu, Jing-Jing Sun, Zhen Tian, Jiang Li

**Affiliations:** Department of Oral Pathology, Shanghai Ninth People’s Hospital, College of Stomatology, Shanghai Jiao Tong University School of Medicine, National Center for Stomatology, National Clinical Research Center for Oral Diseases, Shanghai Key Laboratory of Stomatology, Research Unit of Oral and Maxillofacial Regenerative Medicine, Chinese Academy of Medical Sciences, Shanghai, China

**Keywords:** MNTI, clinicopathological features, *BRAF*, mutation, recurrence

## Abstract

**Purpose:**

To investigate the clinicopathological features and *BRAF* V600E mutation of melanotic neuroectodermal tumor of infancy (MNTI).

**Materials and Methods:**

Eleven cases of MNTI diagnosed at the Department of Oral Pathology were collected. Clinicopathological characteristics were obtained from the medical records. Immunostaining was performed by immunohistochemistry (IHC). Amplification-Refractory Mutation System-qPCR (ARMS-qPCR) and Sanger Sequencing were used to detect *BRAF* V600E mutation.

**Results:**

Of the 11 cases, 3 cases were female and 8 cases were male. The mean age of the first symptoms was 3.2 months (range: 1 to 6 months). Ten cases (90.9%) located in maxilla but only one (9.1%) in mandible. Most of the cases demonstrated well-defined mass with lytic bone destruction and tooth germ affecting radiologically. Histologically, MNTI was consisted of large polygonal melanin-producing epithelioid cells and small round neuroblast-like cells which arranged in irregular alveolar, tubuloglandular and fissured architecture. The epithelioid cells expressed Vim, Pan-CK, NSE and HMB45, while the smalls cells expressed Syn, NSE and scattered Vim. Most cases showed low Ki-67 index (range: <1% to 50%). None of the MNTI cases showed *BRAF* V600E mutation. Most cases were treated with enucleation (45.4%) or curettage (36.4%). Among the 11 cases, 6 cases had follow-up information, and 2 cases had recurrence lesions after surgery.

**Conclusion:**

MNTI, an extremely rare tumor, mainly affects male infants with strong preference for maxilla. Distinct histopathological features and immunohistochemical profile are helpful to distinguish from other melanin-containing tumors and small round cell tumors. No *BRAF* V600E mutation in MNTI is detected in the present study and needs further investigations. The factors that contribute to the local recurrence of MNTI are controversial, but the close follow-up for the patients is recommended.

## Introduction

Melanotic neuroectodermal tumor of infancy (MNTI) is a rare neoplasm which most commonly arises in the anterior region of the maxilla and always develops during the first year of life (1). It was proposed to be of neural crest origin by Borello and Gorlin (2). Later, Hoshino et al. reported high serum levels of catecholamines in MNTI which suggested a neural crest origin of the tumor (3). The clinical manifestations of MNTI include rapid growth, light blue or pink coloration and displacement of developing teeth. MNTI predominantly affects craniofacial regions in more than 90% cases (4). The largest systematic review of 472 cases showed that 62.2% cases involved maxilla, followed by the skull (15.6%), and mandible (7.8%). MNTI shows a slight male predilection with a 1.27:1 male: female ratio (5). On a computed tomography (CT) scan, MNTI always presents as well-defined hyperdense mass. It shows radiolucent lytic lesion within bone and might have local bone destruction features (6). Although MNTI is generally regarded as a benign tumor, most of the lesions shows aggressive clinical behaviors, including rapidly expanding, destruction of surrounding bony tissues and potential of local recurrence.

Morphologically, MNTI consists of two types of cells with distinct features and different immunohistochemical profile (6). More importantly, only few studies had reported molecular alterations in MNTI, including a case with *BRAF* V600E mutation (7), and another case with *CDKN2A* mutation and RPLP1-C19MC gene fusion (8).

Because of the rarity of MNTI, most of the literature have documented single case. We present here with 11 additional cases of MNTI. The clinicopathological characteristics, immunohistochemical profile and molecular alterations, i.e. *BRAF* V600E mutation, of MNTI are analyzed in this study.

## Materials and Methods

### Ethics Statement

The study was approved by the Ethics Committee of Shanghai Ninth People’s Hospital, College of Stomatology, Shanghai Jiao Tong University School of Medicine.

### Patients Selection

Eleven cases of MNTI were retrieved from the electronic database of the Department of Oral Pathology, Shanghai Ninth People’s Hospital, Shanghai Jiao Tong University School of Medicine. All the cases were reassessed by two independent pathologists (RHX and CYZ). The clinicopathological characteristics, including sex, age, tumor location, size and follow-up information were obtained (**Table 1**).

**Table 1:** The clinicopathological features of MNTI.

Case No.	Sex	Age of first symptoms (month)	Age at surgery (month)	Location	Largest diameter (cm)	Platelet count (Reference Range, x10`9)	Radiologic findings	Clinical impression	Management	Follow-up (mo)
1	Male	2	5	Maxilla	2.5	200 (98-380)	Well-defined hypodense lesion. A tooth germ was in the lesion	Teratoma/Dentigerous cyst	Enucleation	NA
2	Male	3.5	5	Maxilla	2.0	464 (85-303)	NA	Cyst	Enucleation	NA
3	Female	2	4.5	Maxilla	5.0	389 (101-320)	NA	Cyst	Biopsy	NA
4	Male	1	2	Maxilla	3.0	586 (85-303)	Well-defined cortical osteolytic expansive mass. A tooth germ was in the lesion	LCH/Dentigerous cyst	Enucleation	NED (67)
5	Female	2	25	Maxilla	3.0	288 (101-320)	A round shaped cystic lesion	Benign tumor/Dentigerous cyst	Curettage	Recurrence (77)
6	Male	6	9	Maxilla	2.0	268 (85-303)	NA	Malignant tumor	Curettage	NA
7	Male	5	6	Mandible	3.0	410 (85-303)	A cortical osteolytic lesion with abnormal soft tissue mass	Teratoma/LCH	Curettage	Recurrence (1)
8*	Male	3	45	Maxilla	6.0	165 (125-350)	Postoperative changes with abnormal bone structure	MNTI	Partial resection of maxilla	NED (85)
9	Male	3	4	Maxilla	3.0	345 (125-350)	Ill-defined cortical osteolytic expansive mass, affecting tooth germ	Teratoma	Enucleation	NED (32)
10	Female	2.5	5	Maxilla	3.0	464 (125-350)	A maxillary mass, affecting nasal base	Oral neoplasm	Curettage	NED (7)
11	Male	5	6	Maxilla	2.0	436 (125-350)	Well-defined mass with lytic bone destruction, affecting tooth germ	Cyst	Enucleation	Recent case

NA: not available; LCH: Langerhans cell histiocytosis; NED: no evidence of disease.

*Recurrent case.

### Immunohistochemistry

Formalin-fixed paraffin-embedded (FFPE) samples were cut into 4 μm sections, and were deparaffinized in xylene and rehydrated through graded ethanol series. The immunohistochemistry procedures were performed using the DAKO AutostainerLink 48 (Agilent Technologies, California), according to the manufacturer’s recommendations. All the cases were stained with pancytokeratin (Pan-CK), HMB45, Melan-A, synaptophysin (Syn), chromogranin (CgA), Ki-67, vimentin (Vim), neuron-specific enolase (NSE), S-100 and desmin (Des) (detailed information for all the antibodies were summarized in **Table 2**). Signals were visualized through the AEC+ High Sensitivity Substrate Chromogen kit (K3469, Agilent Technologies, California).

**Table 2:** Detailed information for all the antibodies used in this study.

Antibody	Clone	Dilution	Host	Manufacturer
Pancytokeratin (Pan-CK)	AE1/AE3	1:200	Mouse	MXB Biotechnologies, Fujian, China
Melanoma	HMB45	1:100	Mouse	MXB Biotechnologies, Fujian, China
Melan-A	A103	1:100	Mouse	MXB Biotechnologies, Fujian, China
Synaptophysin (Syn)	SP11	Read-to-Use	Rabbit	Gene Tech (Shanghai) Company Limited
Chromogranin A (CgA)	LK2H10	1:200	Mouse	MXB Biotechnologies, Fujian, China
Ki-67	MX006	1:200	Mouse	MXB Biotechnologies, Fujian, China
Vimentin (Vim)	V9	1:300	Mouse	Gene Tech (Shanghai) Company Limited
Neuron-specific enolase (NSE)	5E2	1:100	Mouse	Gene Tech (Shanghai) Company Limited
S-100	4C4.9	1:200	Mouse	MXB Biotechnologies, Fujian, China
Desmin (Des)	MX046	1:200	Mouse	MXB Biotechnologies, Fujian, China

### Amplification-Refractory Mutation System-qPCR

Amplification-refractory mutation system-qPCR (ARMS-qPCR) was performed using *BRAF* V600E Mutation Detection Kit (SinoMD Gene Technology, Beijing, China) on the CFX96 machine (BioRad, Hercules, CA, USA). The sensitivity of this kit is 97.89%, and it is sensitive enough for the detection of as low as 1% BRAF V600E mutation. Briefly, the fluorescence signal of FAM using primers specific covering *BRAF* V600E region (determined as Ct_m_) and the signal of HEX using internal control primers were collected for each sample. The elevation of the HEX signal suggested that the amplification procedure was reliable. Besides, for each sample, a paired test using primers covering *BRAF* non-V600E region was conducted as amplification control and was determined as Ct_c_. The data analysis was based on the “ΔCt = (Ct_m_-Ct_c_)” method. If ΔCt ≤ 8, the sample was considered as *BRAF* V600E mutation positive sample. If ΔCt > 8, the sample was considered as a negative one. Except the positive control using in the kit, another two cases of Langerhans cell histiocytosis (LCH) which were previously confirmed with *BRAF* V600E mutation were also used as a positive control.

### Sanger Sequencing

Direct Sanger sequencing for *BRAF* V600E mutation was done by SinoMD Gene Technology Co. Ltd. (Beijing, China). Briefly, genomic DNA was extracted from FFPE samples using QIAamp DNA FFPE Tissue Kit (Cat. No. 56404, Qiagen, Hilden, Germany). The DNA was amplified by polymerase chain reaction (PCR), and then sequencing was done by capillary electrophoresis using Applied biosystem 3500 Genetic Analyzer (Waltham, MA, USA) with forward primer 5’-AGACCTCACAGTAAAAATAGGTGA-3’ and reverse primer 5’- CTGATGGGACCCACTCCATC-3’. The results were compared with the wild type *BRAF* sequence (NM_001354609.2). The same two cases of LCH with confirmed *BRAF* V600E mutation were used as a positive control.

## Results

### MNTI Mainly Affected Male Infants With Strong Preference for Maxilla

**Figure 1:**
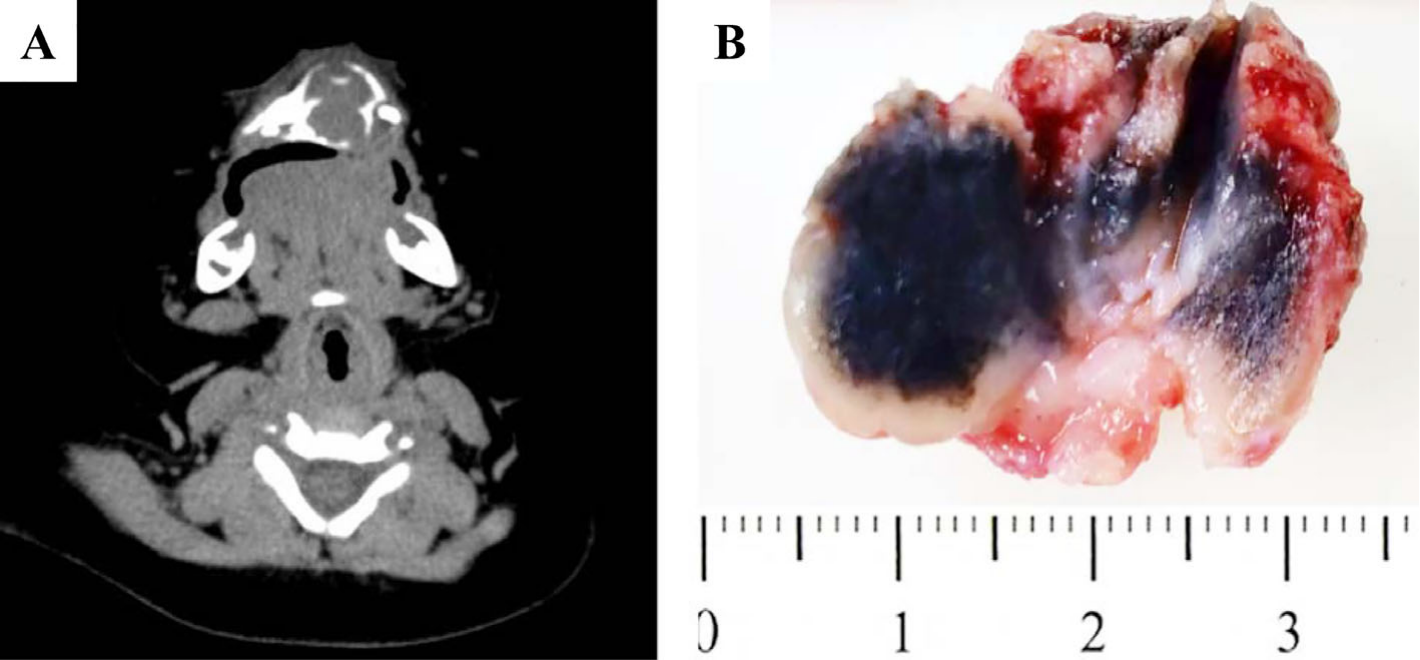
Gross pictures and radiological features of MNTI. **(A)** CT images demonstrated well-defined mass with lytic bone destruction and tooth germ affecting. **(B)** The tumor was firm, unencapsulated and the cut-surface was pigmented.

The detailed clinical information was summarized in **Table 1**. Of eleven cases, three cases were female and eight cases were male. There was a 1:2.67 male predominance. The mean age of the first symptoms was 3.2 months (range: 1 to 6 months). The mean age at diagnosis was 10.6 months (range: 2 to 45 months). One patient was a recurrent case, who had surgery at the age of 45 months (Case 8 in **Table 1**). Ten of eleven cases (90.9%) located in maxilla but only one (9.1%) in mandible. The mean size of tumor was 3.1 cm (range: 2.0 to 6.0 cm). Interestingly, based on the Routine Blood Test, 6 patients (54.5%) showed elevated Platelet Count. Radiologically, most of the cases demonstrated well-defined mass with lytic bone destruction and tooth germ affecting in Computed Tomography (CT) imaging (**Figure 1A**). Based on the clinical and imaging findings, the impression of dentigerous cyst, teratoma, LCH and malignant tumor were suggested before the pathological confirmation.

### MNTI Was Consisted of Two Types of Cells With Distinct Morphological and Immunohistochemical Phenotype

**Figure 2:**
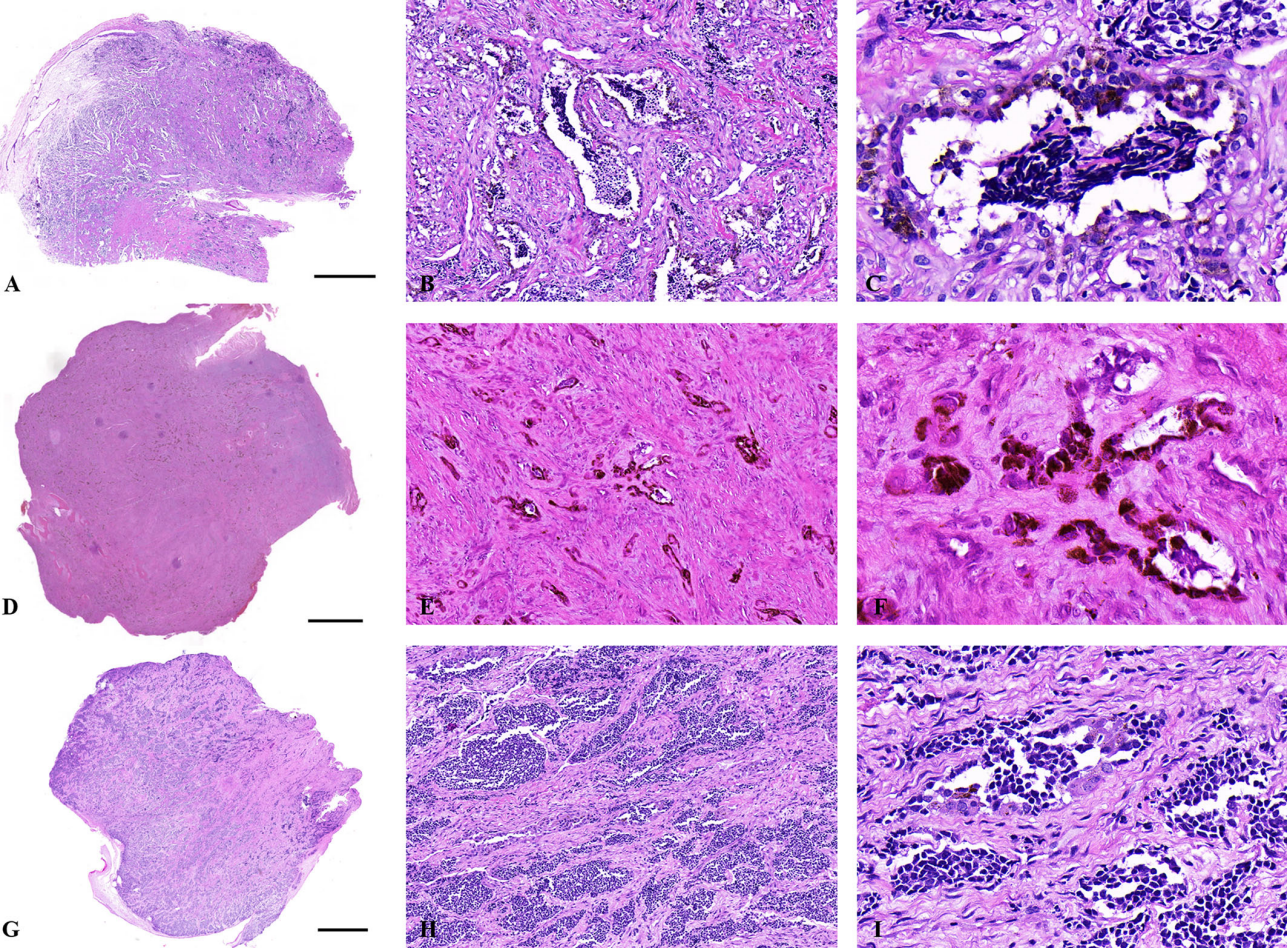
Histological features of MNTIs. **(A–C)** The tumor was consisted of two types of cells which arranged in irregular alveolar, tubuloglandular and fissured architecture, with cords and strands intersected by dense fibroblastic stroma. **(D–F)** this case was predominant with large melanin-producing epithelioid cells. **(G–I)** this case was predominant with small neuroblast-like cells. Hematoxylin-Eosin staining. Scale bar: 2000μm. **(B, E, H)** x100. **(C, F, I)** x400.

Grossly, the tumor was firm, unencapsulated and the cut-surface was pigmented (**Figure 1B**). Morphologically, the tumor was consisted of two types of cells which arranged in irregular alveolar, tubuloglandular and fissured architecture, with cords and strands intersected by dense fibroblastic stroma (**Figure 2**). The large polygonal epithelioid cells were melanin-producing and showed vesicular nuclei, prominent nucleoli and eosinophilic cytoplasm. The small round neuroblast-like cells were surrounded by the epithelioid cells and showed hyperchromatic nuclei and scanty cytoplasm (**Figures 2A–C**). The proportion of the epithelioid cells varied from patient to patient, with a mean of 51% (range: 20% to 95%) (**Table 3**). In some cases, most of the tumor cells were large melanin-producing epithelioid cells (**Figures 2D–F**), while other cases were predominant with small neuroblast-like cells (**Figures 2G–I**).

**Table 3:** The immunohistochemical profile and proportion of epithelioid cells of MNTI.

Case No.	Epithelioid cells						Neurolblast-like cells						S-100, Des, Melan-A	Ki-67 index	The proportion of epithelioid cells
	Pan-CK	HMB45	NSE	Syn	Vim	CgA	Pan-CK	HMB45	NSE	Syn	Vim	CgA			
1	+	+	+	–	+	–	–	–	+	+	All Scattered +	–	All Negative	<1%	30%
2	+	+	+	–	+	–	–	–	+	+		–		<1%	40%
3	+	+	+	–	+	–	–	–	+	+		–		2%	50%
4	+	+	+	–	+	–	–	–	+	+		–		<1%	40%
5	+	+	+	–	+	–	–	–	+	+		–		<1%	50%
6	+	+	+	–	+	–	–	–	+	+		–		<1%	95%
7	+	+	+	–	+	–	–	–	+	+		+		50%	20%
8	+	+	+	–	+	–	–	–	+	+		–		<1%	95%
9	+	+	+	–	+	–	–	–	+	+		+		40%	30%
10	+	+	+	–	+	–	–	–	+	+		+		5%	80%
11	+	+	+	–	+	–	–	–	+	+		+		2%	30%

**Figure 3:**
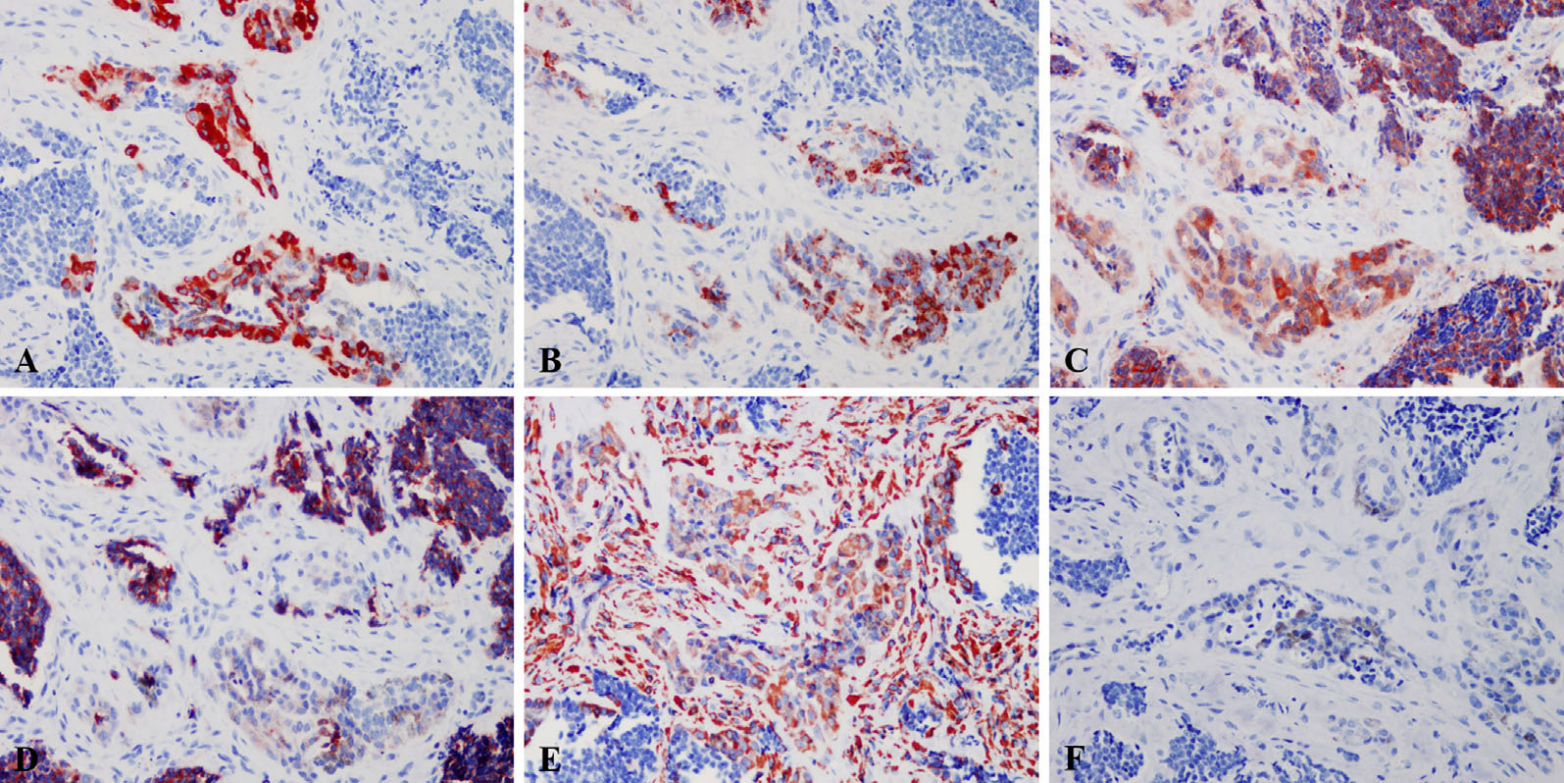
Immunohistochemical profile of MNTI. The epithelioid cells expressed Pan-CK **(A)**, HMB45 **(B)**, NSE **(C)**, while the smalls cells strongly expressed NSE **(C)**, Syn **(D)**. The large cells were also positive for Vim, while the small cells only showed scattered positivity **(E)**. Both large and small cells were negative for S-100 **(F)**. Immunohistochemistry, x400.

Immunohistochemically, the large polygonal epithelioid cell and the small neuroblast-like cells had distinct phenotype. The epithelioid cells of all the tumors expressed Vim, Pan-CK, NSE and HMB45, while the smalls cells expressed Syn (11/11, 100.0%), NSE (11/11, 100.0%) and CgA (4/11, 36.4%). We found that the small neuroblast-like cells showed scattered staining for Vim and all the cases were negative for S-100, Melan-A and Des (**Figure 3**). The Ki-67 index ranged from <1% to 50%. Most of the cases showed low Ki-67 index, i.e. 6 cases (54.5%) with <1%, 2 cases (18.2%) with 2%, 1 case (9.1%) with 5%, 1 case (9.1%) with 40% and 1 case (9.1%) with 50%. The detailed immunohistochemical profile were summarized in **Table 3**.

### None of the MNTI Cases Showed *BRAF* V600E Mutation

**Figure 4:**
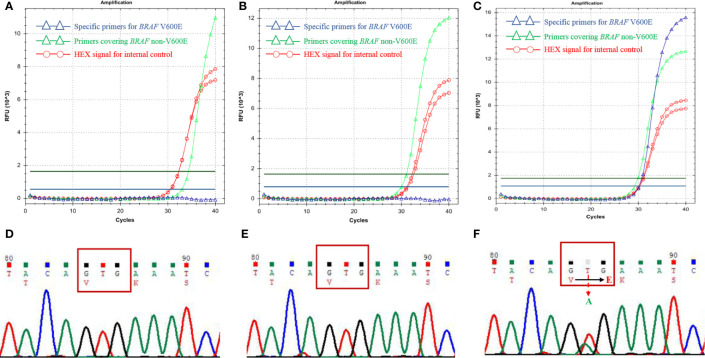
*BRAF* V600E mutation detections in MNTIs and LCH. Detections performed by ARMS-qPCR and Sanger Sequencing showed Case 1 **(A, D)** and Case 11 **(B, E)** were negative for *BRAF* V600E mutation, while the positive control, LCH case was positive for *BRAF* V600E mutation **(C, F)**.

The frequency of *BRAF* V600E mutation was reported as high as 50%-80% in melanoma (9). Based on the similarities between MNTI and melanoma, such as derivation from the neural crest cells and the presence of melanin-producing cells, Gomes et al. (7) suggested that MNTI might also harbor *BRAF* V600E mutation and they found 1 case with *BRAF* V600E mutation. Here we performed ARMS-qPCR and direct Sanger Sequencing in all the 11 cases of MNTI and 2 case of LCH. The latter was used as positive control. All the 11 cases of MNTI did not harbor *BRAF* V600E mutation while 2 cases of LCH were positive for *BRAF* V600E mutation (**Figure 4**).

### Management and Prognosis of MNTI

Of eleven cases, 5 cases were treated with enucleation, 4 cases were treated curettage, the recurrent case were treated with partial resection of maxilla, and one case were performed with biopsy. 6 cases had follow-up information. 2 cases had recurrence lesions one month and 77 months after surgery, respectively. The proportion of the epithelioid cells of those two recurrent cases was 20% and 50% respectively. The Ki-67 index of those two recurrent cases was <1% and 50% respectively. The surgical mode for these two cases which developed recurrence was curettage. The detailed treatment and follow-up information were summarized in **Table 1**.

## Discussion

MNTI is an extremely rare neoplasm occurring frequently in male infant younger than 1-year old. Variable names, including melanotic progonoma (10), pigmented congenital epulis (11), retinal anlage tumor (12), etc. have been used for this tumor since 1918. However, all those synonyms are obsolete and not recommended. The name of MNTI is widely accepted now and the tumor is proposed to be of neural crest origin because the tumor produces VMA (2) and has heterogeneous cellular phenotype microscopically which explains by the mesodermal and ectodermal morphological features displayed by neural crest cells at different stages of their ontogeny (13).

Clinically, MNTI may appear as a rapid expansive, lobulated, but non-ulcerated and painless mass. It always presents with light blue or pink appearance (14). In the current study, MNTI mainly affects the maxilla of male infants, which is consistent with the literature (5, 15). Besides the maxilla, other sites, including skull, mandible (16), and noncranial regions, such as brain, epididymis (2), mediastinum, femur and ovary (4, 17) may be affected. Moreau et al. reported 11 cases of MNTI and found that the mean age of the first symptoms and at diagnosis was 2.8 months and 3.2 months, respectively (15). The current study showed similar results, the mean age of the first symptoms and at diagnosis was 3.2 months and 10.6 months, respectively. It should be noticed that there was a time lag between the onset of symptoms and the diagnosis. The reason for this might be that some patients received surgeries at older age because it was not tolerated at a younger age. One limitation for the current study was that none of the cases were subjected to urinary VMA level detection. We could not acquire this information from this cohort. Interestingly, we identified a proportion of cases in this cohort which showed elevated Platelet Count based on the Routine Blood Test. However, it needed more studies and detections before drawing a reliable conclusion.

MNTI is categorized as one type of benign maxillofacial bone and cartilage tumors in 4^th^ edition of WHO Classification of Head and Neck Tumors. However, radiologically, MNTI always presented with well circumscribed hypodense mass, but may show excessive bone destruction and displacement of dental follicles under CT scan (13). The present case showed a well-defined, unilocular, osteolytic lesion causing expansion and destruction of the maxillary cortical bone, which was in line with the previous publications. The age, tumor location and radiological findings may be suggestive of this tumor. However, we should notice that in the current study, most case were clinically diagnosed with teratoma, cyst or LCH preliminary, only one case were diagnosed as MNTI because this was a recurrent case. Thus, pathological examination is required for a definitive diagnosis.

Late diagnosis might lead to the difficulty in radical resection, because the tumor involves the adjacent structures with rapid invasive growth (4). Once suspected to be MNTI, pathological confirmation might be performed as soon as possible. Histologically, two types of cells with distinct morphological and immunohistochemical phenotype could be seen within this tumor. The tumor cells are arranged in irregular alveolar, tubuloglandular and fissured architecture, with cords and strands intersected by dense fibroblastic stroma. The large melanin-producing polygonal epithelioid cells had vesicular nuclei, prominent nucleoli and eosinophilic cytoplasm, while the small round neuroblast-like cells were surrounded by the epithelioid cells and showed hyperchromatic nuclei and scanty cytoplasm (6). In the present study, the proportion of these two types of cells varied case by case. When a case was predominant with large epithelioid cells (18) or small neuroblast-like cells (19), immunohistochemistry should be performed to confirm the diagnosis because the two type of cells had distinct immunohistochemical phenotype and some of the markers could indicated the presence of the specific type of the cells. In the current study, both large and small cells expressed NSE and Vim, even though that only few small cells expressed Vim. The epithelioid cells were also positive for Pan-CK, HMB45 and the small neuroblast-like cells were positive for Syn. All the tumors were negative for S-100, Melan-A and Des. The immunohistochemical results were in line with the previous publications (6). Those distinct immunohistochemical profile could help distinguishing with other melanin-containing tumors or small round cell tumors, such as melanoma, neuroblastoma, Ewing sarcoma and rhabdomyosarcoma.

The investigations of molecular alterations of MNTI were limited. In 1998, Khoddami et al. (20) studied three typical cases of MNTI and tried to find out the genetic link between MNTI and other small round cell tumors with well-characterized molecular alterations, including *MYCN* gene amplification, deletion of 1p, the presence of the t(11;22)(q24;q12) and the t(11;22)(p13;q12) translocations. Unfortunately, none of the cases yielded positive results. Melanoma showed a high frequency of BRAF V600E mutation. Gomes et al. (7) performed *BRAF* V600E mutation detection in three cases of MNTI based on the hypothesis of the similarities with melanoma. They confirmed 1 case with *BRAF* V600E mutation. Based on those findings, we tried to elucidate the *BRAF* V600E mutation status in the present cohort. As the pigment can interfere with the fluorescence in the qPCR assay, to ensure the accuracy of the detection, internal and amplification control were included in the ARMS-qPCR experiments. Sanger Sequencing method was used to confirm the results, we also included 2 cases of LCH with confirmed *BRAF* V600E mutation as positive control. As the results showed, none of the MNTI cases harbored *BRAF* V600E mutation. The result was inconsistent with Gomes et al.’s study, the tumor enrichment prior to DNA extraction in their investigation might partly explain the mutation detection in one case. However, it had been noticed that another two cases in recent publications were also negative for *BRAF* V600E mutation (18, 21). Paraffin-embedded samples could influence the molecular analysis, further studies on molecular alterations should be conducted using fresh or frozen samples, if possible. Though all those results were based on limited cases, the frequency of *BRAF* V600E mutation in MNTI and the individualized treatment plan that might be developed due to this mutation needed more explorations. It had been reported that besides *BRAF*, other mutated genes, including *CDKN2A*, *NRAS*, *TP53*, *NF1* and *SF3B1* played significant role in different types of melanoma. The results suggested that those genetic alterations might be also candidates for MNTI, and needed further investigations (22). A better understanding of the molecular biology of MNTI is warranted to improve the efficiency of current treatment strategies, especially for those patients with recurrent or metastatic diseases.

At present, the most common treatment protocol is surgery (16, 23). In the present study, enucleation was the predominant treatment (45.4%), followed by curettage (36.4%), partial resection of maxilla (9.1%) and biopsy (9.1%). Chemotherapy and radiation therapy, either alone or in combination with surgery had also been proposed (5, 24). Because MNTI was consisted of large epithelioid cells and neuroblast-like cells, when it was predominant with neuroblast-like cells, chemotherapy has been suggested as the primary treatment mode (25). Adjuvant chemotherapy was also conducted to avoid recurrence after incomplete surgery (19). Targeted therapy should base on the definitive genetic alterations, which had been inconclusive for MNTI until now.

Unfortunately, the biological behavior of MNTI is hard to be predicted by histopathological features, or immunohistochemical markers. Age of diagnosis (5), gender (4, 5), localization in the maxilla, and R1/R2 resection (4), curettage (16), predominance of a neuroblast-like cell component (26) and Ki-67 index (14) were considered as indicators for high risk of local recurrence. However, those results were controversial because a recent publication reported that none of these factors seemed to be associated with a higher risk of recurrence (15). In the present study, it seemed that only the surgical mode, i.e. curettage showed a correlation with the local recurrence. However, the results in the present study should be considered with caution and a lager sample size of patients were needed to confirm our results and allow statistically relevant conclusions to be drawn.

## Conclusion

MNTI, although an extremely rare tumor, it should be added to the differential diagnosis of pediatric population with rapidly growing head and neck lesions. It mainly affects male infants younger than 1-year old with strong preference for maxilla. Distinct histopathological features and immunohistochemical profile are helpful to distinguish from other melanin-containing tumor and small round cell tumors. *BRAF* V600E mutation in MNTI is very rare and needs further investigations. The factors that contribute to the local recurrence of MNTI are controversial, but the close follow-up for the patients are recommended.

## Data Availability Statement

The original contributions presented in the study are included in the article/supplementary material. Further inquiries can be directed to the corresponding authors.

## Ethics Statement

The studies involving human participants were reviewed and approved by Ethics Committee of Shanghai Ninth People’s Hospital, College of Stomatology, Shanghai Jiao Tong University School of Medicine. Written informed consent for participation was not provided by the participants’ legal guardians/next of kin because this is a retrospective study and all the data are anonymous, and the requirement for written consent was therefore waived.

## Author Contributions

Study conception and design: JL and ZT. Acquisition of data: RHX and CYZ. Analysis and interpretation of data: ZT and JJS. Drafting and revising the article: RHX, LZW, and YHH. All authors contributed to the article and approved the submitted version.

## Funding

This work was supported by the National Natural Science Foundation of China (81872187 and 81702694), Postdoctoral Program of Shanghai Ninth People’s Hospital and the SHIPM-mu fund No. JC201901 from Shanghai Institute of Precision Medicine, Ninth People’s Hospital Shanghai Jiao Tong University School of Medicine and CAMS Innovation Fund for Medical Sciences (CIFMS) (Project No. 2019-I2M-5-037). The funders had no role in study design, data collection, analysis and interpretation, decision to submit the article for publication, or preparation of the manuscript.

## Conflict of Interest

The authors declare that the research was conducted in the absence of any commercial or financial relationships that could be construed as a potential conflict of interest.
